# A systematic review on Nipah virus: global molecular epidemiology and medical countermeasures development

**DOI:** 10.1093/ve/veae048

**Published:** 2024-07-25

**Authors:** Foo Hou Tan, Asif Sukri, Nuryana Idris, Kien Chai Ong, Jie Ping Schee, Chong Tin Tan, Soon Hao Tan, Kum Thong Wong, Li Ping Wong, Kok Keng Tee, Li-Yen Chang

**Affiliations:** Department of Medical Microbiology, Faculty of Medicine, Universiti Malaya, Kuala Lumpur, Wilayah Persekutuan 50603, Malaysia; Department of Biological Sciences and Biotechnology, Faculty of Science and Technology, Universiti Kebangsaan Malaysia, Bangi, Selangor 43600, Malaysia; Department of Medical Microbiology, Faculty of Medicine, Universiti Malaya, Kuala Lumpur, Wilayah Persekutuan 50603, Malaysia; Universiti Malaya Medical Centre, Kuala Lumpur, Wilayah Persekutuan 50603, Malaysia; Department of Biomedical Sciences, Faculty of Medicine, Universiti Malaya, Kuala Lumpur, Wilayah Persekutuan 50603, Malaysia; Universiti Malaya Medical Centre, Kuala Lumpur, Wilayah Persekutuan 50603, Malaysia; Department of Medicine, Faculty of Medicine, Universiti Malaya, Kuala Lumpur, Wilayah Persekutuan 50603, Malaysia; Universiti Malaya Medical Centre, Kuala Lumpur, Wilayah Persekutuan 50603, Malaysia; Department of Medicine, Faculty of Medicine, Universiti Malaya, Kuala Lumpur, Wilayah Persekutuan 50603, Malaysia; Department of Biomedical Sciences, Faculty of Medicine, Universiti Malaya, Kuala Lumpur, Wilayah Persekutuan 50603, Malaysia; Universiti Malaya Medical Centre, Kuala Lumpur, Wilayah Persekutuan 50603, Malaysia; Department of Pathology, Faculty of Medicine, Universiti Malaya, Kuala Lumpur, Wilayah Persekutuan 50603, Malaysia; Department of Social Preventive Medicine, Faculty of Medicine, Universiti Malaya, Kuala Lumpur, Wilayah Persekutuan 50603, Malaysia; Department of Medical Microbiology, Faculty of Medicine, Universiti Malaya, Kuala Lumpur, Wilayah Persekutuan 50603, Malaysia; Department of Medical Microbiology, Faculty of Medicine, Universiti Malaya, Kuala Lumpur, Wilayah Persekutuan 50603, Malaysia

**Keywords:** Nipah virus, disease outbreaks, vaccine development, animal models, antivirals

## Abstract

Nipah virus (NiV) is an emerging pathogen that causes encephalitis and a high mortality rate in infected subjects. This systematic review aimed to comprehensively analyze the global epidemiology and research advancements of NiV to identify the key knowledge gaps in the literature. Articles searched using literature databases, namely PubMed, Scopus, Web of Science, and Science Direct yielded 5,596 articles. After article screening, 97 articles were included in this systematic review, comprising 41 epidemiological studies and 56 research developments on NiV. The majority of the NiV epidemiological studies were conducted in Bangladesh, reflecting the country’s significant burden of NiV outbreaks. The initial NiV outbreak was identified in Malaysia in 1998, with subsequent outbreaks reported in Bangladesh, India, and the Philippines. Transmission routes vary by country, primarily through pigs in Malaysia, consumption of date palm juice in Bangladesh, and human-to-human in India. However, the availability of NiV genome sequences remains limited, particularly from Malaysia and India. Mortality rates also vary according to the country, exceeding 70% in Bangladesh, India, and the Philippines, and less than 40% in Malaysia. Understanding these differences in mortality rate among countries is crucial for informing NiV epidemiology and enhancing outbreak prevention and management strategies. In terms of research developments, the majority of studies focused on vaccine development, followed by phylogenetic analysis and antiviral research. While many vaccines and antivirals have demonstrated complete protection in animal models, only two vaccines have progressed to clinical trials. Phylogenetic analyses have revealed distinct clades between NiV Malaysia, NiV Bangladesh, and NiV India, with proposals to classify NiV India as a separate strain from NiV Bangladesh. Taken together, comprehensive OneHealth approaches integrating disease surveillance and research are imperative for future NiV studies. Expanding the dataset of NiV genome sequences, particularly from Malaysia, Bangladesh, and India will be pivotal. These research efforts are essential for advancing our understanding of NiV pathogenicity and for developing robust diagnostic assays, vaccines and therapeutics necessary for effective preparedness and response to future NiV outbreaks.

## 1. Introduction

**Figure 1.**
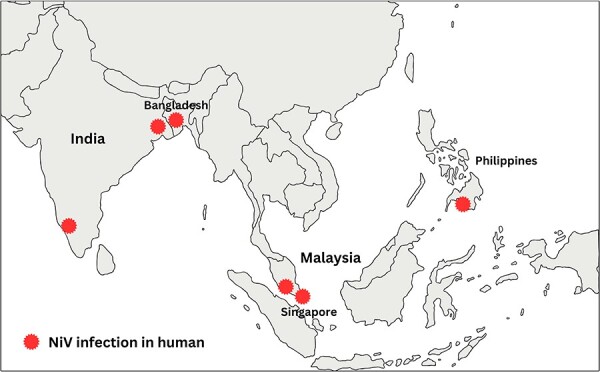
Map indicating reported cases of NiV infection in humans.

Nipah virus (NiV) is a zoonotic emerging virus with high pathogenicity and mortality in human (Chua et al. 2000). First detected in Malaysia in 1998, it has since been detected in other countries such as Bangladesh, India, and Philippines (Fig. 1) (Hsu et al. 2004, Chadha et al. 2006, Gurley et al. 2007a, Ching et al. 2015). NiV causes febrile encephalitis in infected individuals and currently no treatment or vaccine is available (WHO Nipah Virus 2023). *Pteropus* bats are the natural reservoir host for NiV and there is a potential risk of spillover from bats to humans because the areas where these bats live are populated by more than 2 billion people (Epstein et al. 2020, WHO Nipah Virus 2023). NiV spillover events or the zoonotic transmission of NiV from bats to humans are most evident in Bangladesh, which experiences almost annual NiV outbreaks (Nikolay et al. 2019). Moreover, deforestation, the expansion of plantations and farms for poultry use, and land development for urbanization have contributed to the loss of habitats among wildlife, including bats, thus increasing the risk of NiV transmission from bats to humans (Keesing et al. 2010). Transmission of NiV to humans occurs through direct or close contact with bats, commonly through the consumption of date palm sap contaminated with bat secretions and excretions (Luby et al. 2006, Gurley et al. 2017). Transmission of the virus to humans could also occur when in close contact with NiV-infected patients (Chadha et al. 2006, Arunkumar et al. 2019, Banerjee et al. 2019). These NiV transmission routes have been reported in outbreaks in Bangladesh and India. In Malaysia, the transmission route of NiV to humans was primarily through direct or close contact with an intermediate host animal that is infected with NiV (Chua et al. 2000, Goh et al. 2000). Infection of the intermediate host animal could occur from consumption of fruits contaminated with bat secretions and excretions or direct contact with the bat secretions and excretions. Although NiV outbreaks have been limited to a few countries, the World Health Organization (WHO) has identified NiV as a pathogen urgently requiring research and development because of its high pathogenicity in humans (70–100% fatality rate) (Nipah Research and Development (R&D) Roadmap 2023). It is therefore important to better understand the origins and mechanisms of virus transmission, its pathogenicity, and the host’s immune response to facilitate effective public health interventions during NiV outbreaks and to develop effective medical countermeasures.

In this study, we conducted a systematic literature review on the epidemiology and research developments of NiV, including outbreak reports, transmission, clinical symptoms, diagnostic methods, mortality rates, as well as phylogenetic, vaccine, and therapeutic research. Additionally, we identified knowledge gaps in peer-reviewed articles to enhance NiV epidemiology and research development efforts.

## 2. Materials and methods

Literature search was conducted from August 2023 to April 2024. Articles were screened using the Preferred Reporting Items for Systematic Reviews and Meta-Analyses (PRISMA) guidelines. The literature databases used were PubMed, Scopus, Web of Science, and Science Direct. Two researchers independently searched for eligible articles. If there was a disagreement in inclusion and exclusion of the articles in this review, the third researcher was consulted to reach consensus. The search was restricted to full text, peer-reviewed articles published in journals. The following search keyword string was used: (“Nipah virus”) AND (“epidemiology” OR “prevalence” OR “incidence” OR “transmission” OR “risk factor” OR “gene” OR “evolution” OR “vaccine” OR “treatment” OR “antiviral” OR “animal model”).

Inclusion and exclusion criteria of the articles for this systematic review were conducted using PICOT (population, intervention, context, outcome, and timeline) guideline as follows:

- Population: Inclusion, epidemiological studies on human populations worldwide related to NiV. This includes studies focusing on high-risk populations, including those involved in the pig farming industry, close contacts of NiV patients, individuals who handled and consumed raw date palm saps, individuals exposed to fruit bats, and healthcare workers. Additionally, studies on vaccine and therapeutic development are included. Exclusion criteria, epidemiological studies solely involving animals and studies focusing on laboratory investigation into the pathogenesis of NiV, animal model investigations, and molecular biology.
- Intervention: Inclusion, studies that measured antibody titers of NiV, studies that included suspected NiV patients based on clinical findings, studies that confirmed NiV infection using culture and molecular method, and studies investigating the efficacy of vaccines or therapeutics. Exclusion, studies that did not report diagnostic methods for NiV infection, studies that did not mention clinical findings of suspected NiV patients, or *in silico* studies that designed vaccines or therapeutics without subsequent animal studies.
- Context: Inclusion, this systematic review included all observational studies and research development studies, namely outbreak report, cross-sectional studies, intervention studies, cohort studies, case-control studies, case series, longitudinal prevalence studies, seroprevalence studies, prevalence surveys, phylogenetic studies, vaccines studies, and therapeutics studies. All studies must be published in English. Exclusion, studies not published in English, reviews, book chapters, conference abstracts, and letters to the editor.
- Outcome: Seroprevalence, outbreak reports, risk factors, clinical findings, phylogenetic studies, and vaccine and therapeutic development.
- Timeline: No restriction to the time when articles were published.

The data for epidemiological studies were extracted, including the authors, year of publication, country, specific region of the study, type of study, number of samples, type of samples, and epidemiological results. For research development studies, the extracted data included authors, year of publication, source of genomic data, phylogenetic results, animal models, types of vaccines or therapeutics, strain of NiV used, and animal challenge results. All extracted data were arranged using Microsoft Excel 2016 software.

## 3. Results

### 3.1 Screening and quality assessment of studies

**Figure 2.**
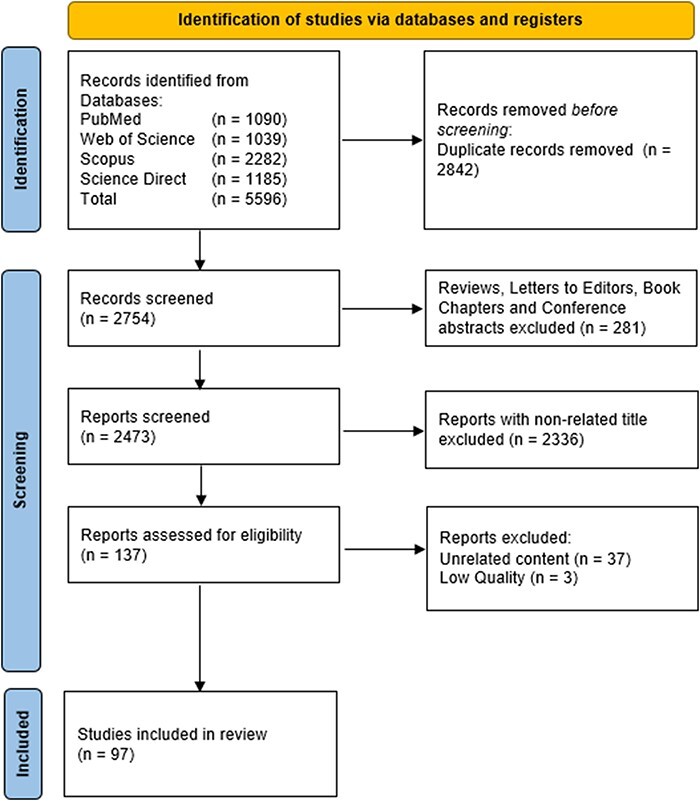
PRISMA flow diagram for the screening process of ninety-seven studies included in this systematic review (Page et al. 2021).

As shown in Fig. 2, our literature search yielded 5596 results (PubMed: 1090, Web of Science: 1039, Scopus: 2282, and Science Direct: 1185). A total of 2842 records were duplicates and excluded from the analysis. Additional 281 records were further excluded because they were reviews, letters to editor, book chapters, or conference abstracts. After the title screening, 2336 records were excluded because they were not relevant to our research question. Thus, 137 articles were eligible for full-text screening. We found 37 articles that were unrelated to our research question and were excluded from our systematic review analysis. The remaining articles were then critically appraised and evaluated using the established guidelines for case-control, qualitative research, case series, and seroprevalence studies (JBI 2023). Two researchers scored the articles based on the guidelines and if there was disagreement, it was resolved by consulting the third researcher prior to consensus. To ensure high-quality studies are included in this review, only the articles that scored ≥50% were included in this systematic review. Three articles failed the assessment process (score less than 50%) and were excluded from this review. Thus, 97 articles were included in this systematic review. Overall, majority of the studies (58; 59.8%) included in this systematic review scored ≥80% in quality check assessment and 22 studies (22.7%) scored ≥70%. The rest of the studies scored ≥60% (*n* = 15) and 50% (*n* = 2).

### 3.2 Characteristics of included studies

A total of 97 articles included in this systematic review were published from 1999 to 2024, with most of it published in 2019 (Nikolay et al. 2019, Arunkumar et al. 2019, Kumar et al. 2019, Thomas et al. 2019, Lo et al. 2019, Mire et al. 2019, Van Doremalen et al. 2019, Yadav et al. 2019, Li et al. 2019, Anderson et al. 2019) (*n* = 10) (Tables 1 and 2), followed by 2000 (Amal et al. 2000, Chew et al. 2000, Chua et al. 2000, Goh et al. 2000, Harcourt et al. 2000, Parashar et al. 2000, Premalatha et al. 2000), 2001 (Chan et al. 2001, Chua et al. 2001, Harcourt et al. 2001, Mounts et al. 2001, Sahani et al. 2001, Wong et al. 2001, Chong et al. 2001a), 2020 (Cappelle et al. 2020, Chandni et al. 2020, Mire et al. 2020, Pallivalappil et al. 2020, Yong et al. 2020, Lo et al. 2020a, 2020b), and 2022 (Foster et al. 2022, Gao et al. 2022, Ithinji et al. 2022, Ramachandran et al. 2022, Van Doremalen et al. 2022, Welch et al. 2022, Yadav et al. 2022) (*n* = 7 each). Other studies were published in 2013 (Mire et al. 2013, Pallister et al. 2013, Ploquin et al. 2013, Sazzad et al. 2013, Wacharapluesadee et al. 2013, Yoneda et al. 2013) and 2023 (De Wit et al. 2023, Monath et al. 2023, Welch et al. 2023, Woolsey et al. 2023, Lu et al. 2023a, 2023b) (*n* = 6 each), 2006 (Chadha et al. 2006, Georges-Courbot et al. 2006, Guillaume et al. 2006, Luby et al. 2006, Weingartl et al. 2006) and 2016 (Chakraborty et al. 2016, Debuysscher et al. 2016, Hegde et al. 2016, Islam et al. 2016, Wacharapluesadee et al. 2016) (*n* = 5 each), followed by 2010 (Freiberg et al. 2010, Rahman et al. 2010, Homaira et al. 2010a, 2010b), and 2024 (Cortes-Azuero et al. 2024, De Campos et al. 2024, Dsouza and Chellasamy 2024, Pastor et al. 2024) (*n* = 4 each), 2004 (AbuBakar et al. 2004, Guillaume et al. 2004, Hsu et al. 2004), 2008 (Hossain et al. 2008, McEachern et al. 2008, Montgomery et al. 2008), 2009 (Bossart et al. 2009, Drexler et al. 2009, Luby et al. 2009), and 2014 (Debuysscher et al. 2014, Geisbert et al. 2014, Lo et al. 2014) (*n* = 3 each). The remaining studies were published in 1999 (Chua et al. 1999, Paton et al. 1999), 2002 (Chan et al. 2002, Chong et al. 2002), 2007 (Gurley et al. 2007a, 2007b), 2012 (Bossart et al. 2012, Rahman et al. 2012), 2015 (Ching et al. 2015, Prescott et al. 2015), 2018 (Dawes et al. 2018, Hassan et al. 2018), 2021 (Geisbert et al. 2021, Rahman et al. 2021) (*n* = 2 each), 2005 (Harcourt et al. 2005), 2011 (Arankalle et al. 2011), and 2017 (Walpita et al. 2017) (*n* = 1 each).

**Table 1.** Characteristics of forty-one NiV epidemiological studies included in this systematic review.

Year of sample collection	Country	Type of study	Positive case	Sample size (excluding control)	Control	Diagnostic test	Author/year
1999	Malaysia	Outbreak investigation	1	28	20	Test not mentioned, HeV antigen was used	Premalatha et al. 2000
1999	Malaysia	Outbreak investigation	7	668	N/A	ELISA (IgG, IgM) against HeV antigen	Sahani et al. 2001
1999	Malaysia	Outbreak investigation	52	69	31	ELISA (IgG, IgM) against HeV antigen	Amal et al. 2000
1999	Malaysia	Outbreak Investigation	94	110	N/A	ELISA (IgG, IgM) against HeV antigen	Goh et al. 2000
1999	Malaysia	Outbreak investigation	110^a^	109	344	ELISA (IgG, IgM) against HeV antigen	Parashar et al. 2000
1999	Malaysia	Outbreak investigation	194	194	N/A	ELISA (IgG, IgM) against HeV antigen	Chong et al. 2001a
1999	Malaysia	Outbreak report	1	1	N/A	ELISA (IgG), RT-PCR	Wong et al. 2001
1999	Malaysia	Outbreak report	3	3	N/A	ELISA (IGM), immunofluorescence against HeV antigen	Chua et al. 1999
1999	Malaysia	Seroprevalence study	3^b^	363	288	ELISA (IgG, IgM) against HeV antigen	Mounts et al. 2001
1999	Singapore	Outbreak investigation	13	13	41	ELISA (IgG, IgM), RT-PCR, Immunohistochemistry	Chew et al. 2000
1999	Singapore	Outbreak report	11	35	N/A	ELISA (IgG, IGM) against HeV antigen, RT-PCR	Paton et al. 1999
1999	Singapore	Seroprevalence study	22	1469	N/A	ELISA (IgG, IgM), RT-PCR	Chan et al. 2002
2001	India	Outbreak investigation	10	18	N/A	ELISA (IgG, IgM), RT-PCR	Chadha et al. 2006
2004	Bangladesh	Outbreak investigation	12	12	36	ELISA (IgM)	Montgomery et al. 2008
2004	Bangladesh	Outbreak investigation	36	210	204	ELISA (IgG, IgM), RT-PCR	Gurley et al. 2007a
2004	Bangladesh	Seroprevalence study	2	105	N/A	ELISA (IgG, IgM)	Gurley et al. 2007b
2007	Bangladesh	Outbreak investigation	7	13	21	ELISA (IgM)	Homaira et al. 2010a
2007	Bangladesh	Outbreak investigation	8	19	24	ELISA (IgG, IgM)	Homaira et al. 2010b
2008	Bangladesh	Outbreak investigation	10	10	40	ELISA (IgG, IgM), qRT-PCR	Rahman et al. 2012
2010	Bangladesh	Outbreak investigation	16	328	58	ELISA (IgM)	Sazzad et al. 2013
2014	Philippines	Outbreak report	17	17	N/A	ELISA (IgM), qRT-PCR	Ching et al. 2015
2018	India	Outbreak investigation	3	279	N/A	ELISA (IgG, IgM)	Kumar et al. 2019
2018	India	Outbreak investigation	12	12	N/A	qRT-PCR	Chandni et al. 2020
2018	India	Outbreak investigation	18	147	N/A	RT-PCR	Thomas et al. 2019
2018	India	Outbreak investigation	23	23	N/A	ELISA (IgG, IgM) against HeV antigen, qRT-PCR	Arunkumar et al. 2019
2018	India	Outbreak investigation	23	23	N/A	qRT-PCR	Pallivalappil et al. 2020
2019	India	Outbreak investigation	1	49	N/A	ELISA (IgG, IgM)	Ramachandran et al. 2020
2021	India	Outbreak report	1	1	N/A	ELISA (IgG, IgM), qRT-PCR	Yadav et al. 2022
1998–99	Malaysia	Outbreak investigation	103	103	N/A	ELISA (IgM) against HeV antigen	Chong et al. 2002
2001, 2003	Bangladesh	Outbreak investigation	25	25	83	ELISA (IgG, IgM)	Hsu et al. 2004
2001–04	Bangladesh	Outbreak investigation	92	92	N/A	ELISA (IgG, IgM), RT-PCR	Hossain et al. 2008
2001–07	Bangladesh	Outbreak investigation	122	122	N/A	ELISA (IgG, IgM)	Luby et al. 2009
2001–14	Bangladesh	Outbreak investigation	248	248	N/A	ELISA (IgG, IgM)	Nikolay et al. 2019
2004–05	Bangladesh	Outbreak investigation	12	124	33	ELISA (IgG, IgM)	Luby et al. 2006
2004–12	Bangladesh	Outbreak investigation	157	157	632	ELISA (IgM)	Hegde et al. 2016
2010–11	Bangladesh	Outbreak investigation	43	43	155	ELISA (IgG, IgM)	Chakraborty et al. 2016
2011–14	Bangladesh	Outbreak investigation	14	14	N/A	ELISA (IgG, IgM)	Islam et al. 2016
2013–14	Bangladesh	Outbreak investigation	16	335	N/A	ELISA (IgM), qRT-PCR	Hassan et al. 2018
2014–16	Cambodia	Seroprevalence study	0	418	N/A	ELISA (IgG)	Cappelle et al. 2020
Not mentioned	Malaysia	Outbreak investigation	20	26	N/A	ELISA (IgM) against HeV antigen	Chua et al. 2001
Not mentioned	Malaysia	Seroprevalence study	19	177	N/A	ELISA (IgG)	Yong et al. 2020

a 80/109 tested positive, another 30 tested positive from control group.

b Likely to be false positive

N/A: not applicable

**Table 2.** Characteristics of fifty-six NiV research development studies included in this systematic review.

Vaccine/antiviral/monoclonal antibody	Type of study	Source of NiV isolates/animal model	Authors/year
N/A	Phylogenetic study	Human, pig, cat, dog	Chua et al. 2000
N/A	Phylogenetic study	Human	Harcourt et al. 2000
N/A	Phylogenetic study	Human	Chan et al. 2001
N/A	Phylogenetic study	Human	Harcourt et al. 2001
N/A	Phylogenetic study	Human, flying fox, pig	AbuBakar et al. 2004
N/A	Phylogenetic study	Human	Harcourt et al. 2005
N/A	Phylogenetic study	Bat	Drexler et al. 2009
N/A	Phylogenetic study	Human, pig, bat	Rahman et al. 2010
N/A	Phylogenetic study	Human, pig, bat	Arankalle et al. 2011
N/A	Phylogenetic study	Human, pig, bat	Wacharapluesadee et al. 2013
N/A	Phylogenetic study	Human, pig, bat	Wacharapluesadee et al. 2016
N/A	Phylogenetic study	Human, pig, bat	Anderson et al. 2019
N/A	Phylogenetic study	Human, pig, bat	Li et al. 2019
N/A	Phylogenetic study	Human, pig, bat	Yadav et al. 2019
N/A	Phylogenetic study	Human, pig, bat	Rahman et al. 2021
N/A	Phylogenetic study	Human, bat, pig, dog	Cortes-Azuero et al. 2024
N/A	Phylogenetic study	Human, flying fox, pig, dog	De Campos et al. 2024
N/A	Phylogenetic study	Human	Dsouza et al. 2024
Recombinant vaccinia virus	Vaccine study	Syrian hamster	Guillaume et al. 2004
Canarypox virus vector	Vaccine study	Landrace pig	Weingartl et al. 2006
Subunit vaccine	Vaccine study	Domestic short hair cat	McEachern et al. 2008
Subunit vaccine	Vaccine study	African green monkey	Bossart et al. 2012
rVSV	Vaccine study	Ferret	Mire et al. 2013
Subunit vaccine	Vaccine study	Ferret	Pallister et al. 2013
rAdV	Vaccine study	Syrian hamster	Ploquin et al. 2013
rMeV	Vaccine study	Syrian hamster	Yoneda et al. 2013
rVSV	Vaccine study	Syrian hamster	Debuysscher et al. 2014
rVSV	Vaccine study	Syrian hamster	Lo et al. 2014
rVSV	Vaccine study	African green monkey	Prescott et al. 2015
rVSV	Vaccine study	Syrian hamster	Debuysscher et al. 2016
VLP	Vaccine study	Syrian hamster	Walpita et al. 2017
rAdV	Vaccine study	Syrian hamster	Van Doremalen et al. 2019
rVSV	Vaccine study	African green monkey	Mire et al. 2019
mRNA vaccine	Vaccine study	Syrian hamster	Lo et al. 2020b
Subunit vaccine	Vaccine study	African green monkey	Geisbert et al. 2021
rAdV	Vaccine study	African green monkey	Van Doremalen et al. 2022
rVSV	Vaccine study	African green monkey	Foster et al. 2022
Subunit vaccine	Vaccine study	Mouse	Gao et al. 2022
rVSV	Vaccine study	Syrian hamster	Ithinji et al. 2022
DNA vaccine, rAdV	Vaccine study	Syrian hamster	Lu et al. 2023
DNA vaccine, rAdV	Vaccine study	Syrian hamster	Lu et al. 2023
rVSV	Vaccine study	African green monkey	Monath et al. 2023
Non-spreading virus replicon particle	Vaccine study	Syrian hamster, mice	Welch et al. 2023
rVSV	Vaccine study	African green monkey	Woolsey et al. 2023
Recombinant anti-CD40 antibody	Vaccine study	African green monkey	Pastor et al. 2024
Ribavirin, Poly(I)-Poly(C12U), 6-Aza-uridine	Therapeutics study	Syrian hamster	Georges-Courbot et al. 2006
Monoclonal antibody	Therapeutics study	Syrian hamster	Guillaume et al. 2006
Monoclonal antibody	Therapeutics study	Ferret	Bossart et al. 2009
Ribavirin, Chloroquine	Therapeutics study	Syrian hamster	Freiberg et al. 2010
Monoclonal antibody	Therapeutics study	African green monkey	Geisbert et al. 2014
Favipiravir	Therapeutics study	Syrian hamster	Dawes et al. 2018
Remdesivir	Therapeutics study	African green monkey	Lo et al. 2019
Griffithsin	Therapeutics study	Syrian hamster	Lo et al. 2020a
Monoclonal antibody	Therapeutics study	Ferret	Mire et al. 2020
Defective interfering particles (DIP)	Therapeutics study	Syrian hamster	Welch et al. 2022
Remdesivir	Therapeutics study	African green monkey	De Wit et al. 2023

N/A: not applicable.

Among the 41 studies involving NiV epidemiological studies, 16 studies were conducted in Bangladesh, 12 in Malaysia, 8 in India, 3 in Singapore, and 1 each from Cambodia (Cappelle et al. 2020) and Philippines (Ching et al. 2015). Samples used in 28 studies were collected in a single year, while samples from 11 studies were collected over multiple years, and there was no information on the sampling time for two studies. Among the studies that collected samples in a single year, 12 studies collected samples in 1999 during the NiV outbreak in Malaysia (*n* = 9) and Singapore (*n* = 3), followed by eight studies from India that collected samples in 2018 (*n* = 5), 2001, 2019, and 2021 (*n* = 1 each) during the NiV outbreaks. Seven studies from Bangladesh collected samples in 2004 (*n* = 3), 2007 (*n* = 2), and 2008 and 2019 (*n* = 1 each), while only one study from Philippines collected samples in 2014 (Ching et al. 2015). Meanwhile, the 11 studies which samples were collected over multiple years were conducted in Bangladesh (*n* = 9), Malaysia (*n* = 1), and Cambodia (*n* = 1). Both studies that did not provide information on sampling time were conducted in Malaysia (*n* = 2). The types of studies carried out included outbreak investigation (*n* = 31), outbreak report (*n* = 5), and seroprevalence study (*n* = 5). The samples collected were primarily from NiV patients (confirmed and probable cases), close contacts of NiV patients, high-risk individuals who worked in pig farms, palm juice workers, individuals who lived near to the habitats of fruit bats, and individuals without a history of NiV exposure as control subjects. The sample size, the number of positive NiV patients, and the number of controls described are shown in Table 1.

The 56 studies on NiV research developments focused on phylogenetic studies (*n* = 18), vaccine development (*n* = 27), and therapeutics development (*n* = 11). In phylogenetic studies, NiV isolated from humans was most extensively studied (*n* = 17), followed by isolates from pigs and bats (*n* = 12 each), with the remaining studies investigating NiV from dogs (*n* = 3) and cats (*n* = 1). For vaccine development, most studies used the NiV strain from the Malaysia outbreak for virus challenges in the animal models (*n* = 12), followed by the strain from Bangladesh (*n* = 8). The remaining studies used both strains for the challenge (*n* = 3), one study utilized Nipah pseudovirus (Gao et al. 2022), while three studies did not specify the strain used. In therapeutics development studies, the NiV strain from Malaysia was predominantly used (*n* = 8), while the remaining studies used the strain from Bangladesh (*n* = 3). The sources of NiV isolates in the phylogenetic studies, the animal models used in vaccines and therapeutics studies, and the types of vaccines and therapeutics developed are detailed in Table 2.

### 3.3 NiV epidemiology

#### 3.3.1 Seroprevalence of NiV

Of the 41 epidemiological studies, five studies reported seroprevalence of NiV infection that targeted human populations at risk of exposure to NiV; two of these studies were conducted in Malaysia, and one each in Singapore (Chan et al. 2002), Bangladesh (Gurley et al. 2007b) and in Cambodia (Cappelle et al. 2020). The first study of NiV seroprevalence in Malaysia was conducted among healthcare workers (HCWs) exposed to patients hospitalized with outbreak-related encephalitis after 1 March 1999 (Mounts et al. 2001). At least three samples in the cohort (1.02%) were tested positive for NiV IgG antibodies by enzyme-linked immunosorbent assay (ELISA) using Hendra virus (HeV) antigens. However, these three samples were likely false positives because no IgM response was detected and they were negative for anti-NiV neutralizing antibodies. The second study of NiV seroprevalence in Malaysia, reported more recently among Orang Asli (indigenous people), found that 10.73% of the study cohort tested positive for antibodies against NiV nucleoprotein (N) (Yong et al. 2020). Although the presence of anti-NiV neutralizing antibodies in these positive samples was not determined, the significantly higher seroprevalence observed among adults and higher seroprevalence among those with domestic animals indicate important exposure risk factors to NiV. In Singapore, the seroprevalence study was conducted after the NiV outbreak in March 1999 among various risk groups potentially exposed to NiV (Chan et al. 2002). The study reported 1.50% of the cohort (*n* = 22) to have detectable antibodies to NiV by ELISA using HeV antigens and NiV antigens. Twenty of the samples had NiV IgM antibodies, and all these samples had detectable NiV IgG antibodies except one sample because the individual died before the appearance of detectable IgG. The remaining two samples were only positive for NiV IgG antibodies, which were later confirmed positive for NiV because of the presence of detectable anti-NiV neutralizing antibodies. All positive cases were male abattoir workers. In Bangladesh, seroprevalence study was performed on HCWs who had exposure to at least one NiV patient (Gurley et al. 2007b). A total of 105 HCWs participated in the study, including 26 physicians, 68 nurses, and 11 cleaners or other patient attendants. Two of the participants tested positive for IgG against NiV but not in IgM, indicating a likely infection from a previous outbreak. Some of the participants (*n* = 12) experienced febrile illness during the study period in March 2004. However, none of the participants tested positive for IgM against NiV. Among the 105 HCWs, <30% of them used personal protective equipment while providing care to NiV patients; however, no correlation between use of personal protective equipment and NiV transmission from patient to HCWs was observed in this study. In Cambodia, seroprevalence studies were conducted from 2013 to 2016 among workers potentially exposed to fruit bats (*Pteropus lylei*), such as palm juice collectors, sellers, and bat hunters (Cappelle et al. 2020). Two cohorts were reported: one with 164 participants from 41 households in Kandal and the other with 254 participants from 103 households in Battambang. None of the participants were tested positive for NiV antibodies.

#### 3.3.2 Diagnosis of NiV infection

Samples collected for diagnosis of NiV infection included sera, whole blood, cerebrospinal fluid (CSF), oral and throat swabs, urine, brain tissue, and endotracheal aspirates. Among the studies included in the epidemiological section of this systematic review, 31.71% (13/41) reported the use of HeV antigen for detecting NiV antibodies with ELISA, immunofluorescence assay (IFA), and immunohistochemistry assay (IHC). Many of the studies utilizing HeV antigen in diagnostic assays were conducted during the early stages of the NiV outbreak when specific diagnostic reagents for NiV had not yet been established. These studies were reported in Malaysia (*n* = 10), Singapore (*n* = 2), and India (*n* = 1). Nine studies, including those from Malaysia (*n* = 6), Singapore (*n* = 2) and India (*n* = 1), reported the use of HeV antigen in ELISA to detect both IgM and IgG from serum or CSF in NiV patients. Three studies from Malaysia utilized HeV antigen in ELISA for detecting only IgM in the serum or CSF of NiV patients. In another study, HeV antigen was utilized to detect the presence of antibodies against NiV, although the specific test method was not described (Premalatha et al. 2000). Conversely, in a separate study from Malaysia, HeV antibodies were used in an indirect IFA to detect the presence of NiV in infected cells (Chua et al. 1999). HeV antibodies were also used in a study from Singapore to detect NiV in the brain tissue of one deceased patient with IHC (Chew et al. 2000). When specific antigens and antibodies for NiV became available, studies (25/41) reported their use in ELISA and IHC. Among the 25 studies that utilized NiV reagents in ELISA to diagnose NiV infection, 16/25 (64%) assayed for the presence of both IgM and IgG, 6/25 (24%) assayed for the presence of NiV IgM only, and 3/25 (12%) assayed for NiV IgG only. In one study from Singapore, a NiV-specific antibody was used to detect the presence of NiV in the brain tissue of a deceased patient (Chan et al. 2002).

Reverse transcription polymerase chain reaction (RT-PCR) and real-time quantitative RT-PCR (qRT-PCR) assays were developed to specifically detect the viral genome in various NiV patient samples, including CSF, throat swab, serum, urine, and saliva. In this systematic review, 8 out of 41 (19.51%) studies utilized RT-PCR for NiV detection, while 7 out of 41 (17.07%) utilized qRT-PCR for NiV diagnosis. The NiV N gene was amplified with RT-PCR from patients’ sample in four studies conducted in Bangladesh (*n* = 2), Malaysia (*n* = 1), and India (*n* = 1). The targeted gene in RT-PCR was not specified in the four remaining studies from Singapore (*n* = 3) and India (*n* = 1). Among the seven studies that used qRT-PCR for NiV diagnosis, only three studies, conducted Bangladesh (*n* = 2) and India (*n* = 1), reported the use of a Taqman probe targeting the NiV N gene. In two additional studies from India, the usage of qRT-PCR targeting the N gene was described, but not the specific type of qRT-PCR used. The remaining two studies from India and the Philippines reported using qRT-PCR for NiV detection without specifying the type of qRT-PCR used or its target gene.

Among the 41 epidemiological studies analyzed in this systematic review, only six reported attempting NiV isolation from collected samples. However, successful NiV isolation was achieved in just four studies, where the virus was isolated NiV from various patient samples, including CSF, throat swab, nasal swab, and urine (Chua et al. 1999, 2001, Chew et al. 2000, Hossain et al. 2008). Two studies from India, conducted during the outbreaks in 2001 and 2021, were unsuccessful in NiV isolation (33.33%; 2/6). Among the successfully isolated NiV cases reported in the four studies, two studies from Malaysia observed the formation of multinucleated giant syncytia in infected Vero cells during the 1999 outbreak. The other two studies from Singapore and Bangladesh successfully isolated NiV during the 1999 and 2004 outbreaks, respectively.

#### 3.3.3 Transmission of NiV

The transmission of NiV from person-to-person has been reported in 23 studies, primarily during NiV outbreaks in Bangladesh (*n* = 14), India (*n* = 7), Malaysia (*n* = 1), and the Philippines (*n* = 1). NiV transmission was identified to have occurred during direct contact with NiV-infected corpse, close contact with NiV patients, and in hospital settings as a nosocomial infection (Chadha et al. 2006, Gurley et al. 2007a, Sazzad et al. 2013). However, some studies from Bangladesh (*n* = 1), India (*n* = 1), and Malaysia (*n* = 1) reported little to no risk of person-to-person transmission, as all individuals who had contact with NiV patients tested negative for NiV infection (Mounts et al. 2001, Gurley et al. 2007b, Ramachandran et al. 2022). Another investigated mode of NiV transmission to humans involved direct or close contact with infected animals, specifically pigs (*n* = 13), bats (*n* = 7), cows (*n* = 1), and horses (*n* = 1). In the studies reporting on the NiV outbreak in Malaysia (*n* = 10) and Singapore (*n* = 3), direct or close contact with infected pigs predominantly occurred during activities such as feeding and cleaning pigs on the pig farm, as well as during the slaughtering process in the abattoir (Amal et al. 2000, Chew et al. 2000, Chan et al. 2002, Chong et al. 2002). Findings from the studies in Bangladesh (*n* = 3) and India (*n* = 2), noted that exposure to bats also posed a risk of NiV transmission (Luby et al. 2009, Pallivalappil et al. 2020, Yadav et al. 2022). However, in studies conducted in Malaysia (*n* = 1) and Cambodia (*n* = 1) concerning NiV transmission through bats, none of the exposed individuals tested positive for NiV (Cappelle et al. 2020, Yong et al. 2020). While a case-control study in Bangladesh observed a correlation between NiV patients and their exposure to a sick cow, the lack of NiV testing in the cow raises the possibility that this correlation might be coincidental (Hsu et al. 2004). Conversely, in the Philippines, direct or close contact with infected horses during slaughter and the consumption of horsemeat were identified as the mode of NiV transmission (Ching et al. 2015). In Bangladesh, alongside person-to-person transmission of NiV, the virus was reported to be transmitted through the consumption of NiV-contaminated palm juice (*n* = 7).

#### 3.3.4 Signs and symptoms of NiV infection

There were 32 studies of the 41 epidemiological studies included in this systematic review that reported the signs and symptoms of NiV infection. The common signs and symptoms of NiV infection reported in studies from Malaysia (*n* = 7) and Singapore (*n* = 4) included fever, cough, headache, febrile illness, and reduced consciousness. At least four studies from Malaysia and Singapore (*n* = 2 each) reported severe symptoms of NiV infection, such as encephalitis, pneumonia, and coma or loss of consciousness. Encephalitis was also reported in India (*n* = 1) and the Philippines (*n* = 1), while loss of consciousness was reported in six studies from Bangladesh. Apart from the signs and symptoms mentioned, additional symptoms including difficulty in breathing, altered mental status and vomiting were commonly reported in studies from Bangladesh (*n* = 13), India (*n* = 7), and Philippines (*n* = 1). Furthermore, two studies from India reported acute respiratory distress symptoms.

Signs and symptoms of NiV spreaders were described in two studies from Bangladesh. The definition of NiV spreader is a NiV patient who had close contact with at least one person who developed NiV illness within 5–15 days after contact. In both studies, the NiV spreaders were severely ill patients with cough and difficulty to breathe, which potentially increased the likelihood of spreading the virus. In one of the studies, all NiV spreaders had cough and difficulty in breathing, and all of them did not survive the disease (Luby et al. 2009). The mortality rate of the NiV spreaders in the other study was not reported. However, NiV RNA was detected in the oral secretions of all the NiV patients who did not survive, suggesting a possible relationship between NiV virulence and tissue tropism (Hassan et al. 2018).

The shortest incubation time before onset of illness was eight days (Ching et al. 2015), while the longest was 17 days (Luby et al. 2006). The mean incubation time before onset of illness was 10 days as reported in five studies from Bangladesh, four studies from India, and one study from Philippines. For Malaysia, only one study reported incubation time of 14 days or less in most of the patients (Goh et al. 2000). Average days of death after onset of illness was 5–6 days as reported in 10 studies from Bangladesh and two studies from India. While for Malaysia, the average days of death after onset of illness was 10 days as reported in three studies. Asymptomatic cases were only reported in four studies from Malaysia and two studies from Singapore, while no asymptomatic cases were reported in Bangladesh and India.

#### 3.3.5 Mortality rate of NiV

The mortality rate of NiV was reported in 25 studies, specifically 13 studies from Bangladesh, five from India, four from Malaysia, two from Singapore, and one study from the Philippines. The mortality rate ranged from 7.7% (1/13 NiV patients) in Singapore (Chew et al. 2000) to 92% (11/12 patients) in Bangladesh (Luby et al. 2006). Two studies from Singapore and one study from Bangladesh (Hassan et al. 2018) reported mortality rate below 30%. Four studies from Malaysia reported a mortality rate of 30–40%. Five studies reported mortality rates of 40–70% and these studies were from Bangladesh (*n* = 4) and the Philippines (*n* = 1) (Ching et al. 2015). The remaining 13 studies reported mortality rates greater than 70%, and these studies were from Bangladesh (*n* = 8) and India (*n* = 5). Among these 13 studies, 10 studies reported the mortality rate of NiV to range from 70–80% to 80–90% (*n* = 5 each), while three studies reported the mortality rate of above 90%.

#### 3.3.6 Risk factors for NiV infection

The investigated risk factors for NiV infection encompassed variables, such as age, gender, relationship with NiV patients, contact with NiV patients, exposure to NiV-infected animals, and exposure to NiV-contaminated palm juice. Among the 41 epidemiological studies, studies from Malaysia and Bangladesh (*n* = 1 each) reported age-related risk factors. The study in Malaysia reported an increased risk of NiV infection among adults compared to children (Yong et al. 2020), while the study in Bangladesh indicated a higher infectivity rate in children (Montgomery et al. 2008). Regarding gender-related risk factors, six studies from Bangladesh (*n* = 3), Malaysia (*n *= 2), and India (*n* = 1) provided insights. In studies from Bangladesh (*n* = 3) and India (*n* = 1), slightly higher infection rates were reported in males compared to females (50–60%) (Luby et al. 2009, Nikolay et al. 2019, Thomas et al. 2019). Similarly, the studies in Malaysia (*n* = 2) showed a significantly higher rate of infections in males (>80%) (Amal et al. 2000, Goh et al. 2000). While the correlation between gender and other factors was not studied, it is noteworthy that the majority (80.8%) of patients in the study from Malaysia were employed in the pig farming industry (Amal et al. 2000). Additionally, two studies from Bangladesh reported a higher risk for the female spouses of NiV-infected male patients to contract NiV (Homaira et al. 2010a, Nikolay et al. 2019).

Close contact with NiV patients is a significant risk factor for NiV infection, as reported in Bangladesh (*n* = 11), India (*n* = 6), and Malaysia (*n* = 2). Another identified risk factor for NiV infection was exposure to NiV-infected animals, specifically pigs (*n* = 9 from Malaysia, *n* = 3 from Singapore), cows (*n* = 1 from Bangladesh), and horses (*n* = 1 from the Philippines). Additionally, at least seven studies from Bangladesh reported that the consumption of contaminated palm juice posed a risk for NiV, although one study reported the lack of correlation between NiV infection and palm fruit-related activities (Montgomery et al. 2008).

### 3.4 NiV research developments

#### 3.4.1 NiV phylogenetic studies

Phylogenetic analysis was conducted in 18 studies included in this systematic review. These studies included sampling from humans, pigs, bats, dogs, and cats and were conducted in Malaysia (*n* = 6), Bangladesh (*n* = 3), India, Thailand (*n* = 2 each), and Africa (*n* = 1). The remaining four studies performed phylogenetic analysis using data obtained from available databases. Most studies compared NiV gene sequences across multiple species, including humans, pigs, bats, dogs, and cats (*n* = 12). Five studies focused exclusively on human NiV gene sequences, and one study analyzed NiV gene sequence solely from bats (Drexler et al. 2009). Among the 18 studies, three analyzed the full genome of NiV, while nine focused on a single NiV gene, specifically the N gene (*n* = 6) or the L gene (*n* = 3). Five studies analyzed both the full genome of NiV and six NiV genes (N, P, M, F, G, and L), while the remaining study analyzed only six genes of NiV (N, P, C, M, F, and G) (Harcourt et al. 2000).

After the initial NiV outbreak in Malaysia, three phylogenetic studies compared NiV sequences with other paramyxoviruses (Harcourt et al. 2000, 2001, Chan et al. 2001). These studies highlighted a high similarity between NiV and HeV, with both viruses clustering distinctly apart from other paramyxoviruses. This distinct clustering led to the formation of a new genus known as *Henipavirus*, with NiV and HeV identified as its members (Harcourt et al. 2001). In India, a study conducted in Kerala collected samples from human and bats, and found that NiV sequences isolated from a human patient were most closely related to the local bat samples rather than to other NiV strains (Yadav et al. 2019). Similarly, a separate study in Bangladesh reported similar findings, indicating a local spillover event of NiV from bats to humans (Rahman et al. 2021). Recent studies comparing full genome sequences of NiV and NiV N gene sequences available in databases further demonstrated the formation of a distinct clade for NiV sequences from India (De Campos et al. 2024, Dsouza and Chellasamy 2024). These studies proposed that NiV strains from India should be classified as new strain separate from NiV strains found in Bangladesh. However, the NiV sequence from Kerala, India, differed from the strain found in West Bengal, India, which was reported to be highly similar to the NiV strain from Bangladesh (Arankalle et al. 2011). Furthermore, the evolution of NiV over time was analyzed and described in four studies, suggesting that the most recent common ancestor of NiV emerged around the 1940s (Li et al. 2019, Rahman et al. 2021, De Campos et al. 2024, Cortes-Azuero et al. 2024). The NiV Malaysian strain then diverged around the 1980s, followed by NiV Bangladesh strain around the 2000s.

#### 3.4.2 NiV vaccine development

A total of 27 studies focused on NiV vaccine research and development were identified and included in this systematic review. A diverse range of vaccine platforms were used, which were specifically of 11 types. These platforms included recombinant vesicular stomatitis virus (rVSV) (*n* = 10), subunit vaccines, recombinant adenovirus (rAdV) (*n* = 5 each), DNA vaccines (*n* = 2), recombinant vaccinia virus, mRNA vaccine, recombinant anti-CD40 antibody, virus-like particles (VLP), non-spreading virus replicon particles, canarypox virus vector, and recombinant measles virus (rMeV) (*n* = 1 each). The majority of studies evaluated a single type of vaccine (*n* = 25), although two studies compared DNA vaccines with recombinant adenovirus vaccines. Most studies utilized single antigen vaccine candidates (*n* = 19), while six studies tested vaccines with two antigens, and two studies examined vaccines with three antigens. The NiV G protein was the most commonly studied antigen (*n* = 19), followed by the NiV F protein (*n* = 9), HeV G protein (*n* = 5), NiV N protein, and the entire NiV particle (*n* = 2 each). Specific strains of NiV used as vaccine candidates were often not reported, though six studies used proteins from the NiV Bangladesh strain, and one study developed VLP from NiV Malaysian strain (Walpita et al. 2017). The majority of studies tested vaccines on a single type of animal model (*n* = 25), with two studies using two different animal models for NiV challenge. Syrian hamsters were the most frequently used animal model (*n* = 13), followed by African green monkeys (*n* = 10), mice, ferrets (*n* = 2 each), domestic short hair cats, and landrace pigs (*n* = 1 each).

Among these NiV vaccine studies, the majority reported complete protection against NiV in vaccinated groups, with no survival observed in unvaccinated groups. However, in 10 studies, some unvaccinated animals survived a lethal dose of NiV challenge. Among these, six studies used the NiV Malaysian strain, three did not specify the NiV strain, and one used NiV Bangladesh strain (Lu et al. 2023b). Nine studies reported only partial protection for some vaccinated groups. Factors that limited the protective capability of the vaccines included low vaccination dosage and short intervals between vaccination and viral challenge (Debuysscher et al. 2016, Foster et al. 2022, Monath et al. 2023, Welch et al. 2023). Despite extensive research into NiV vaccines, there has been only two clinical trials of a NiV vaccine, with one began in 2022 utilizing mRNA vaccine [Dose Escalation, Open-Label Clinical Trial to Evaluate Safety, Tolerability and Immunogenicity of a Nipah Virus (NiV) MRNA Vaccine, MRNA-1215, in Healthy Adults] and another in early 2024 utilizing recombinant adenovirus as the vaccine candidate (First-in-Human Vaccine Trial for Deadly Nipah Virus Launched).

#### 3.4.3 NiV therapeutics development

Among the 11 studies related to NiV therapeutics included in this review, seven focused on antivirals, and four on monoclonal antibodies. The antivirals studied included ribavirin, remdesivir (*n* = 2 each), poly(I)-poly(C12U), 6-azauridine, chloroquine, favipiravir, griffithsin, and defective interfering particles (DIP) (*n* = 1 each). The majority of the studies examined a single antiviral agent (*n* = 9), although one study tested ribavirin and chloroquine in combination (Freiberg et al. 2010), and another tested ribavirin, poly(I)-poly(C12U), and 6-azauridine (Georges-Courbot et al. 2006). Regarding NiV monoclonal antibody therapy, two studies focused on antibodies targeting the NiV G receptor binding site, one focused on NiV F (Mire et al. 2020), and one utilized antibodies against both NiV G and NiV F (Guillaume et al. 2006).

In the review of 11 antiviral studies, Syrian hamsters were the most commonly used animal model (*n* = 6), followed by African green monkeys (*n* = 3), and ferrets (*n* = 2). The majority of these studies used the NiV Malaysian strain for challenge experiments (*n* = 8), while the remaining studies used the NiV Bangladesh strain (*n* = 3). In most studies, no untreated control animal survived NiV challenge; however, one study reported a 20% survival rate in untreated animals challenged with the NiV Malaysian strain (Freiberg et al. 2010). In terms of efficacy, most antivirals did not provide complete protection against a lethal dose of NiV challenge. Antiviral agents that demonstrated full protection against lethal NiV challenge included favipiravir (Dawes et al. 2018) and remdesivir (Lo et al. 2019). Favipiravir, when started on the same day as the viral challenge followed by one daily dose for a total of 2 weeks, provided full protection to the challenged animals. For remdesivir, African green monkeys demonstrated 100% protection with daily dose of 10 mg/kg for 12 days starting from the day of the viral challenge. However, a recent study under similar conditions, where the first dose of the remdesivir was administered three days after challenge, showed only 67% protection (De Wit et al. 2023). These studies highlight the importance of timely antiviral treatment for NiV infection survival.

Monoclonal antibodies were evaluated as NiV therapeutics in several studies with varying levels of protection efficiency. One study reported protection efficiencies of 33% and 100% when antibodies targeting NiV G receptor binding site were administered one day before or immediately after the NiV challenge, respectively (Bossart et al. 2009). Another study using an antibody targeting NiV F found that treatment on the day of the NiV challenge provided the highest survival rate, while protection efficiency decreased significantly with delays in administering the first dose (Guillaume et al. 2006). In terms of dosage, one study treated hamsters challenged with the NiV Malaysian using monoclonal antibodies targeting NiV G on the same day as the challenge but achieved only 75% protection (Guillaume et al. 2006). Another study on monoclonal antibodies targeting NiV G fully protected challenged ferrets with two doses of antibodies administered on days one and three or days three and five after challenge with the NiV Malaysian strain (Mire et al. 2020). However, differences in the animal models used in these studies may have influenced the observed protection efficacy.

Additionally, the passive transfer of antibodies elicited by the vaccines was described in three NiV vaccine development studies. In the first study, antibodies elicited in Syrian hamsters immunized with recombinant vaccinia virus expressing NiV G or NiV F were administered to hamsters challenged with the NiV Malaysian strain (Guillaume et al. 2004). Similarly, another study involved antibodies elicited in Syrian hamsters using rVSV expressing NiV G, NiV F, or NiV N (Debuysscher et al. 2014). Both studies reported full protection against viral challenge with antibodies targeting NiV G and NiV F, but only partial protection with antibodies targeting NiV N. The remaining study evaluated passive antibodies elicited against NiV G using a rAdV in Syrian hamsters challenged with the NiV Bangladesh strain (Van Doremalen et al. 2019). The protection efficiency in this study was observed to be only 83.3%.

## 4. Discussion

Although NiV has been discovered more than 20 years ago, the disease epidemiology and pathogenicity are still not well understood. This knowledge gap is largely attributed to the lack of resources, including reagents and assays for NiV and containment facilities for research, particularly in the countries where the viral disease has been reported (Cloeckaert and Kuchler 2020). Recognizing the significant mortality rate in humans and the potential of NiV to cause future pandemics, the WHO has listed NiV in its research and development blueprint, emphasizing the need for robust diagnostics, vaccines, and therapeutics (Nipah Research and Development (R&D) Roadmap 2023). We systematically reviewed studies on NiV epidemiology from its initial outbreak in Malaysia to date, spanning a period of 25 years. Our aim was to identify and determine key knowledge gaps in the existing body of epidemiological research.

Seroprevalence studies are important to assess past and present exposure to NiV, as well as to provide information on asymptomatic cases among at-risk populations. These studies provide insights into the disease’s prevalence, acting as early indicators of the disease, to enable the strategic planning and implementation of public health measures to control and mitigate the disease. Despite the high mortality rate of NiV and its pandemic potential, a limited number of seroprevalence studies have been conducted among populations with exposure to the virus (Mounts et al. 2001, Chan et al. 2002, Gurley et al. 2007b, Cappelle et al. 2020, Yong et al. 2020). A seroprevalence study in Singapore after the outbreak in 1999 focused on abattoir employees working with pigs (Chan et al. 2002). Given that the pig farming industry was the source of the outbreaks in Malaysia and Singapore, our result is in good agreement with those findings (Paton et al. 1999, Chong et al. 2002). It was reported that all the NiV patients from Malaysia and Singapore during the 1999 outbreak had contact with pigs in their daily lives (Paton et al. 1999, Chong et al. 2002). As for the seroprevalence study in Singapore, all seropositive individuals were abattoir workers exposed to pigs (Chan et al. 2002). Although the antibodies detected could be due to the presence of cross-reactive antibodies to other viruses such as HeV, the seroprevalence results also suggested the possibility of asymptomatic cases of NiV that were undetected in some individuals. At least two studies investigated NiV seroprevalence among HCWs in Malaysia and Bangladesh, which revealed a negligible risk of nosocomial infection with NiV (Mounts et al. 2001, Gurley et al. 2007b). However, person-to-person transmission of NiV was reported in Bangladesh and India, primarily among family members of infected patients (Gurley et al. 2007a, Homaira et al. 2010b, Kumar et al. 2019, Pallivalappil et al. 2020). This likely occurred because the family members spent more time with the patients compared to HCWs. Overall, NiV IgM persisted in NiV survivors for at least three months, and IgG remains detectable even after 10 years of infection (Ramasundrum et al. 2000, Siva et al. 2009). This underscores the robustness of seroprevalence studies.

Other reported seroprevalence studies focused on populations in Malaysia and Cambodia, potentially exposed to bats or having close proximity to previous epidemic sites (Chong et al. 2003, Cappelle et al. 2020, Yong et al. 2020). The findings from these studies reveal a low percentage of seropositive individuals (<10%), suggesting low risk of spillover in these countries. In contrast, Bangladesh experiences frequent NiV outbreaks, with bat exposure identified as a primary contributing factor, attributed to the unique practice of consuming raw date palm, which has the potential to be contaminated by bats (Sahani et al. 2001, Nikolay et al. 2019, Ithinji et al. 2022). Overall, the exposure to and transmission of NiV in the outbreaks in Malaysia, Bangladesh, and India are contextually different. In, Malaysia, transmission of NiV was documented to primarily occur through direct or indirect contact with an intermediate host, specifically pigs that had direct or indirect contact with bat’s secretion and excretion (Chua et al. 1999, 2001, Amal et al. 2000, Goh et al. 2000, Parashar et al. 2000, Premalatha et al. 2000, Sahani et al. 2001, Wong et al. 2001, Chong et al. 2001a, 2002), while in Bangladesh and India, transmission was through consumption of food contaminated with bat’s secretion and excretion, and person-to-person transmission (Hsu et al. 2004, Chadha et al. 2006, Luby et al. 2006, 2009, Gurley et al. 2007a, Hossain et al. 2008, Montgomery et al. 2008, Homaira et al. 2010a, 2010b, Rahman et al. 2012, Sazzad et al. 2013, Chakraborty et al. 2016, Hegde et al. 2016, Islam et al. 2016, Hassan et al. 2018, Arunkumar et al. 2019, Kumar et al. 2019, Nikolay et al. 2019, Thomas et al. 2019, Chandni et al. 2020, Pallivalappil et al. 2020, Yadav et al. 2022). This insight highlights the importance of cultural and regional practices in understanding and addressing the varying risks associated with NiV transmission.

Understanding of the risk factors associated with NiV infection is important for the effective minimization or prevention of its spread. In addition to the previously mentioned risk factors, age was reported as a risk factor for NiV in two studies (Gurley et al. 2007a, Yong et al. 2020). A Malaysian study highlighted that adults have a higher risk of NiV due to their working environment in the pig-farming industry, exposing them constantly to NiV-infected pigs (Yong et al. 2020). In contrast, in Bangladesh, children were identified as having a higher risk for NiV, attributed to childhood activities such as tree climbing and fruit gathering (Gurley et al. 2007a). These activities potentially expose children to environments contaminated with NiV to a greater extent than adults, suggesting the complex relationship between age- and occupational-related factors in NiV transmission.

Timely and accurate diagnosis is important for the early identification of NiV as symptoms and clinical findings can be inconclusive. In this systematic review, ELISA methods were the most frequently used approach for diagnosing NiV (38/41, Table 1). While ELISA can offer specificity and sensitivity, its high labor intensity and the potential for cross reactivity with antibodies from other henipaviruses or paramyxoviruses have been noted (Chua et al. 1999). There is a need for a robust and validated serological assay that is highly specific and sensitive for the diagnostics and surveillance studies of NiV disease. Another diagnostic assay used in the studies analyzed in this systematic review was qRT-PCR, which is highly specific and sensitive, but it requires trained expertise and expensive equipment (Arumugam et al. 2020). Additionally, qRT-PCR is applicable only for detecting the NiV genome during active infection and it is not suitable for seroprevalence studies. In addition to the diagnostic approaches outlined above, brain magnetic resonance imaging (MRI) was used to confirm the diagnosis of NiV patients in Malaysia (Tan et al. 2000). Furthermore, it proved effective in detecting abnormal MRI findings in asymptomatic individuals. Given the high infectivity rate of NiV and cases of asymptomatic infection (Tan et al. 1999), coupled with the frequent outbreaks of NiV in middle- and low-income countries, it is crucial to develop and establish robust and affordable diagnostic assays, such as antigen rapid kits with quick result turnover.

The isolation of NiV for confirming its infection is important as highlighted in several studies included in this systematic review (Chua et al. 1999, 2001, Chew et al. 2000, Chadha et al. 2006, Hossain et al. 2008, Yadav et al. 2022). The success of virus isolation depends on the appropriateness of the clinical specimen type collected and the timing of specimen collection during the infection stage. The collection of clinical specimens at the acute stage is paramount for successful virus isolation. Previous findings indicate that the presence of anti-NiV IgM during later stages of the illness correlates with a decreased viral shedding rate (Chua et al. 2001). Currently, the number of NiV strains successfully isolated remains limited, similarly with information on the viral genome sequence. To date, there are only five full-genome sequences of human NiV documented from the 1998 to 1999 outbreak in Malaysia (National Center for Biotechnology Information B000), and an additional five from the outbreaks in India in 2007 (*n* = 1) and 2018 (*n* = 4). A more extensive dataset is available comprising of 29 full-genome sequences of human NiV from various outbreaks in Bangladesh, from 2004 (*n* = 5), 2008 (*n* = 3), and 2011–15 (*n* = 21). The NiV genome sequences exhibit variations across different geographical locations (Whitmer et al. 2021). Taken together, understanding the NiV genome sequences, particularly from Malaysia and India, could provide valuable insights into how differences in viral strains contribute to the transmission dynamics of the virus. It is imperative to intensify efforts in NiV isolation and genome sequencing from these regions to broaden the dataset on viral strain diversity. This expanded dataset is important for a more comprehensive understanding of viral epidemiology and disease pathogenicity, as well as for the development of vaccines and other medical countermeasures to ensure effective prevention and control of the disease.

The prognosis and mortality rates of NiV varied across countries that reported outbreaks. In Bangladesh and India, a high mortality rate of >70% was documented (Chadha et al. 2006, Luby et al. 2006, 2009, Gurley et al. 2007a, Hossain et al. 2008, Rahman et al. 2012, Sazzad et al. 2013, Chakraborty et al. 2016, Hegde et al. 2016, Arunkumar et al. 2019, Thomas et al. 2019, Chandni et al. 2020, Pallivalappil et al. 2020), while in Malaysia, the mortality was about 40% during the outbreak in September 1998 to April 1999 (Goh et al. 2000, Chong et al. 2002). In contrast, Singapore reported a lower mortality rate, with two studies indicating rates of <10% during the outbreak in March 1999 (Paton et al. 1999, Chew et al. 2000). It is noteworthy that the Singapore outbreak took place in an abattoir that primarily processed imported pigs, mostly from Malaysia (Chew et al. 2000). However, despite this connection, the mortality rate for NiV in Singapore was notably lower than that in Malaysia. This is likely attributed to the Singaporean outbreak being less extensive in scale compared to Malaysia. Moreover, it occurred when effective containment measures had already been implemented during the latter phase of the Malaysian outbreak. On the other hand, the higher mortality rate in Bangladesh and India suggests the NiV strains from these countries may be more virulent compared to NiV strains from Southeast Asia countries, including Malaysia, Singapore, and the Philippines (Mire et al. 2016). Variations in the NiV disease mortality rate among different countries could also be linked to differences in their healthcare systems For example, the percentage of HCWs using personal protective equipment while giving care to NiV patients was lower in Bangladesh compared to Malaysia (Mounts et al. 2001, Gurley et al. 2007b). In addition, host factors, particularly diabetes mellitus, were noted to increase the mortality of NiV patients in Malaysia by 123% (Chong et al. 2001b). Furthermore, relapsed encephalitis was reported in 9% symptomatic NiV survivors and 3.4% of asymptomatic NiV survivors (Tan et al. 2002, Chong and Tan 2003, Sejvar et al. 2007, Abdullah et al. 2012). Some individuals with relapsed encephalitis succumbed to the disease; however, reports on the mortality rate associated with NiV relapse were notably scarce (Tan et al. 2002).

In addition to NiV epidemiological research, the increasing availability of NiV sequences has enabled more phylogenetic studies. These studies suggest that the NiV Malaysia strain diverged from the most recent common ancestor around the 1980s, followed by NiV Bangladesh strain around the 2000s. While NiV infections in Bangladesh typically transmit directly from bats to humans, in Malaysia, infections primarily originate from bats to pigs to humans. This difference in transmission routes likely contributed to the divergence of the NiV Malaysia from NiV Bangladesh. Two recent studies proposed that the NiV strain from India should be considered separate from NiV Bangladesh (De Campos et al. 2024, Dsouza and Chellasamy 2024). In Bangladesh, most NiV infections are linked to the consumption of bat-contaminated date palm sap, whereas in India, person-to-person transmission is more common, with index cases originating from indirect contact with bats or unknown sources (Arunkumar et al. 2019, Thomas et al. 2019, Chandni et al. 2020). Similar to the situation in Malaysia, these differences in transmission routes might lead to NiV India evolving into a unique strain. However, a study conducted in West Bengal, India, reported a sequence highly identical to NiV Bangladesh (Arankalle et al. 2011). This high similarity was expected given West Bengal’s close proximity to Bangladesh. In contrast, the studies suggesting that NiV India should be considered as separate strain compiled sequences from across India, including Kerala, which is geographically farther from Bangladesh than West Bengal. Therefore, in countries with large land areas like India, the location of sampling should be considered for phylogenetic analysis rather than treating all sequences from India as a single viral strain. Moreover, NiV infections occur annually in Bangladesh, particularly during the date palm sap harvest season (Satter et al. 2023), which provides a significant amount of genetic data for NiV Bangladesh. In contrast, outbreaks in Malaysia and India are less frequent. Malaysia experienced only one outbreak in 1998–1999, resulting in limited genetic data for NiV Malaysia. India has had several NiV outbreaks, including two reported in West Bengal and, more recently, three outbreaks in Kerala. Future retrospective studies could analyze samples collected from early NiV outbreaks, during a time when genome sequencing was not readily available, to gain insights into the evolution of NiV.

Given the high mortality rate associated with NiV infection, the development of vaccines and therapeutics is critical. However, the survival of some animals in the experiments involving untreated groups following a lethal dose of NiV raises concerns about the efficacy of these interventions (Weingartl et al. 2006, Freiberg et al. 2010, Yoneda et al. 2013, Prescott et al. 2015, Debuysscher et al. 2016, Walpita et al. 2017, Welch et al. 2023, Woolsey et al. 2023, Lu et al. 2023b). Most studies where unvaccinated individuals survived were challenged with NiV Malaysia strain, possibly indicating lower virulence compared to NiV Bangladesh (Goh et al. 2020). Vaccines developed using the G protein from HeV provided full protection against lethal challenges from both NiV Malaysia and NiV Bangladesh (McEachern et al. 2008, Bossart et al. 2012, Pallister et al. 2013, Geisbert et al. 2021), demonstrating cross protection among *Henipaviruses*. In one study, vaccines developed from NiV Bangladesh protected ferrets against a lethal dose of NiV Malaysia (Mire et al. 2013). On the other hand, the antivirals reviewed in the studies included in this systematic review are broad-spectrum (Rada and Dragúň 1977, Adis 2004, Lee 2019, Geraghty et al. 2021, Shetty and Namachivayam 2021). Specifically, favipiravir (Dawes et al. 2018) and remdesivir (Lo et al. 2019) demonstrated full protection against lethal NiV Malaysia strain and NiV Bangladesh strain viral challenges in animal models, respectively. Both of these antiviral agents target the viral replication process, similar to ribavirin, an antiviral that was administered during the 1999 NiV outbreak in Malaysia, which significantly reduced the mortality rate associated with NiV (Chong et al. 2001a). The findings suggest that NiV vaccines and therapeutics may not be strain specific. To ensure the reliability of vaccines and therapeutics efficacy, future research should consider utilizing the more virulent NiV Bangladesh strain instead of NiV Malaysia. Additionally, comparing the NiV Bangladesh and NiV India strains, which have been reported more recently, could provide valuable insights.

In studies where vaccines and therapeutics provided partial protection, the timing of treatment was critical for survival outcomes. Animals vaccinated less than three days before challenge or post-challenge did not survive in several instances (Debuysscher et al. 2016, Foster et al. 2022, Welch et al. 2023). Conversely, in antiviral studies, the majority of animals survived when treated within 24 hours of NiV challenge, but survival rates dropped significantly with treatments administered earlier or later than this timeframe (Guillaume et al. 2006, Bossart et al. 2009, De Wit et al. 2023). However, in real-world scenarios, administering antiviral treatment within 24 hours of infection is impractical because NiV symptoms typically manifest after at least one week, making effective antiviral intervention too late (Ching et al. 2015). Therefore, vaccination remains the most effective approach in preventing NiV outbreaks.

To date, a notable absence of research exists regarding the knowledge, attitude, and practices related to NiV across diverse cultures and regions. This gap warrants further investigation, as it is important for the development of effective educational initiatives tailored for various communities, especially those at a higher risk of NiV spillover from its natural reservoir host to humans. It is also essential to disseminate information to these communities regarding the signs and symptoms of NiV disease, along with preventive and control measures. Community awareness regarding NiV and fostering appropriate responses are paramount to mitigating the risk of NiV transmission.

## 5. Conclusions

This systematic review provides a comprehensive overview of the global molecular epidemiology, vaccine development, and therapeutics advancements for NiV, highlighting knowledge gaps in the existing literature. The majority of the analyzed epidemiological studies were conducted in Bangladesh, indicating the heightened prevalence of NiV in this country. Variations in transmission patterns, associated with diverse cultural and regional practices across countries, were observed, indicating the multifaceted modes of NiV transmission. Serological assays and molecular tests are common diagnostic tools for identifying NiV infections, while virus isolation was reported with limited success. Presently, the availability of NiV isolates and its genome sequences remains limited, particularly from countries that have experienced NiV outbreaks such as Malaysia and India. Despite shared symptoms in NiV outbreaks across different countries, there is a significant variation in mortality rates, ranging from >70% to <40%. Taken together, these findings highlight the complexity of NiV in various aspects, particularly the spread of the virus, diversity of viral strains and the pathogenicity of the disease. Addressing these complexities is important for the understanding of NiV epidemiology, with the ultimate goal of preventing or minimizing casualties in future outbreaks. The limited number of available seroprevalence studies and the small dataset of viral strain diversity emphasize the need for more extensive seroprevalence studies across diverse populations at risk. Several NiV vaccine studies have demonstrated full protection against NiV infection, however, only two clinical trials began recently in USA and England, countries where NiV infection have never been reported (Dose Escalation, Open-Label Clinical Trial to Evaluate Safety, Tolerability and Immunogenicity of a Nipah Virus (NiV) MRNA Vaccine, MRNA-1215, in Healthy Adults, First-in-Human Vaccine Trial for Deadly Nipah Virus Launched). It is crucial to accelerate the development of additional vaccine candidates for clinical trials, particularly in countries like Bangladesh where NiV infections are frequently reported. Additionally, increased efforts to expand the dataset of NiV genome sequences, particularly in areas with a history of NiV outbreaks and inhabited by *Pteropus* bats, are essential for advancing our understanding and preparedness for future occurrences. Lastly, there should be a greater emphasis on developing vaccines and treatments for NiV to reduce its mortality rate and potentially eradicate the virus altogether.

## Data Availability

Not Applicable.
